# Enhancer of zeste homolog 2-catalysed H3K27 trimethylation plays a key role in acute-on-chronic liver failure via TNF-mediated pathway

**DOI:** 10.1038/s41419-018-0670-2

**Published:** 2018-05-22

**Authors:** Tianhui Zhou, Ye Sun, Ming Li, Yongsen Ding, Rongkun Yin, Ziqiang Li, Qing Xie, Shisan Bao, Wei Cai

**Affiliations:** 1 Department of Infectious Diseases, Ruijin Hospital, Shanghai Jiao Tong University School of Medicine, Shanghai, 200025 China; 2 Department of Infectious Diseases, The Fifth People’s Hospital of Suzhou, Suzhou, 215007 China; 3 Discipline of Pathology, School of Medical Sciences and Bosch Institute, University of Sydney, Sydney, NSW 2006 Australia

## Abstract

Acute-on-chronic liver failure is mainly due to host immunity self-destruction. The histone H3 lysine 27 (H3K27) trimethylating enzyme, enhancer of zeste homolog 2 (EZH2) mediates epigenetic silencing of gene expression and regulates immunity, also involves pathogenesis of several liver diseases. The current study was to determine the role of methyltransferase EZH2 and its catalysed H3K27 trimethylation (H3K27me3) in liver failure, and to further investigate the potential target for liver failure treatment. EZH2 and its catalysed H3K27me3 were determined in peripheral blood mononuclear cells (PBMC) from liver failure patients and Kupffer cells from experimental mice. Furthermore, GSK126 (an inhibitor for EZH2 trimethylation function) was applied in liver failure mice in vivo, and lipopolysaccharide-stimulated mononuclear cells in vitro. EZH2 and H3K27me3 were significantly upregulated in human PBMC from liver failure patients or murine Kupffer cells from the liver failure animals, respectively. GSK126 ameliorated disease severity in liver failure mice, which maybe attribute to down-regulate circulating and hepatic proinflammatory cytokines, especially TNF *via* reducing H3K27me3. In-depth chromatin immunoprecipitation analysis unravelled that decreased enrichment of H3K27me3 on *Tnf* promotor, resulting in TNF elevation in Kupffer cells from liver failure mice. Nuclear factor kappa B (NF-κB) and protein kinase B (Akt) signalling pathways were activated upon lipopolysaccharide stimulation, but attenuated by using GSK126, accompanied with decreased TNF in vitro. In conclusion, EZH2 and H3K27me3 contributed to the pathogenesis of liver failure via triggering TNF and other indispensable proinflammatory cytokines. EZH2 was to modify H3K27me3 enrichment, as well as, activation of the downstream NF-κB and Akt signalling pathways.

## Introduction

Acute-on-chronic liver failure (ACLF) is a severe deterioration of liver function accompanied with massive hepatocyte necrosis/apoptosis and recruitment of hepatic inflammatory cells, leading to multi-organ dysfunction^1^. Despite extensive intervention, the short-term mortality in liver failure patients is unacceptably high, mainly due to inadequate response to conventional treatment and lack of opportunity for liver transplantation^2^. Host immunity, leading to secretion of inflammatory cytokines and activation of excessive immune cascade, are accompanied with massive hepatic injury during the early episodes of ACLF^3^. Inflammatory cytokines, e.g., tumour necrosis factor (TNF), progressively increase in relation to the severity and prognosis of ACLF^4,5^. However, the precise underlying mechanism of ACLF, particularly how these proinflammatory cytokines is regulated by host immunity remains to be explored.

Epigenetics, including DNA methylation and histone modifications, mainly regulate gene expression without altering gene sequence^6^. Emerging evidence suggests that epigenetics play vital roles in modulation of immunity^7,8^. Targeting at epigenetic modifications, novel therapeutic strategy for regulation of immune response during liver failure are definitely urgent for patients suffering from fatal liver failure and multi-organ dysfunction.

The histone H3 lysine 27 (H3K27) trimethylating enzyme, enhancer of zeste homolog 2 (EZH2) is an indispensable histone methyltransferase contributing to silence of the target genes via histone modifications^9^. EZH2-catalysed trimethylation of H3K27 (H3K27me3) is overexpressed in many cancers with poor prognosis^10,11^. EZH2/H3K27me3 also involves in liver diseases, including nonalcoholic fatty liver diseases, liver fibrosis and hepatitis B^12,13^. Furthermore, methyltransferase EZH2 regulates T cells proliferation and differentiation, contributes to autoimmune diseases and alloimmunity^14,15^. However, the role of EZH2/H3K27me3 in host immunity, particularly in regulating inflammation during liver failure remains to be explored.

In the current study, it was investigated the potential role of EZH2/H3K27me3 during the development of liver failure. Moreover, the underlying mechanism involved EZH2-catalysed H3K27me3 in the liver failure was explored at molecular level via modification of methyltransferase activity of EZH2 in vivo. Our data suggest that there was a correlation between increased EZH2/H3K27me3 and upregulated TNF in liver failure. Such data may provide useful information for both basic research and clinical precision medicine with specific target(s).

## Results

### EZH2 and H3K27me3 are significantly increased in ACLF patients

**Fig. 1:**
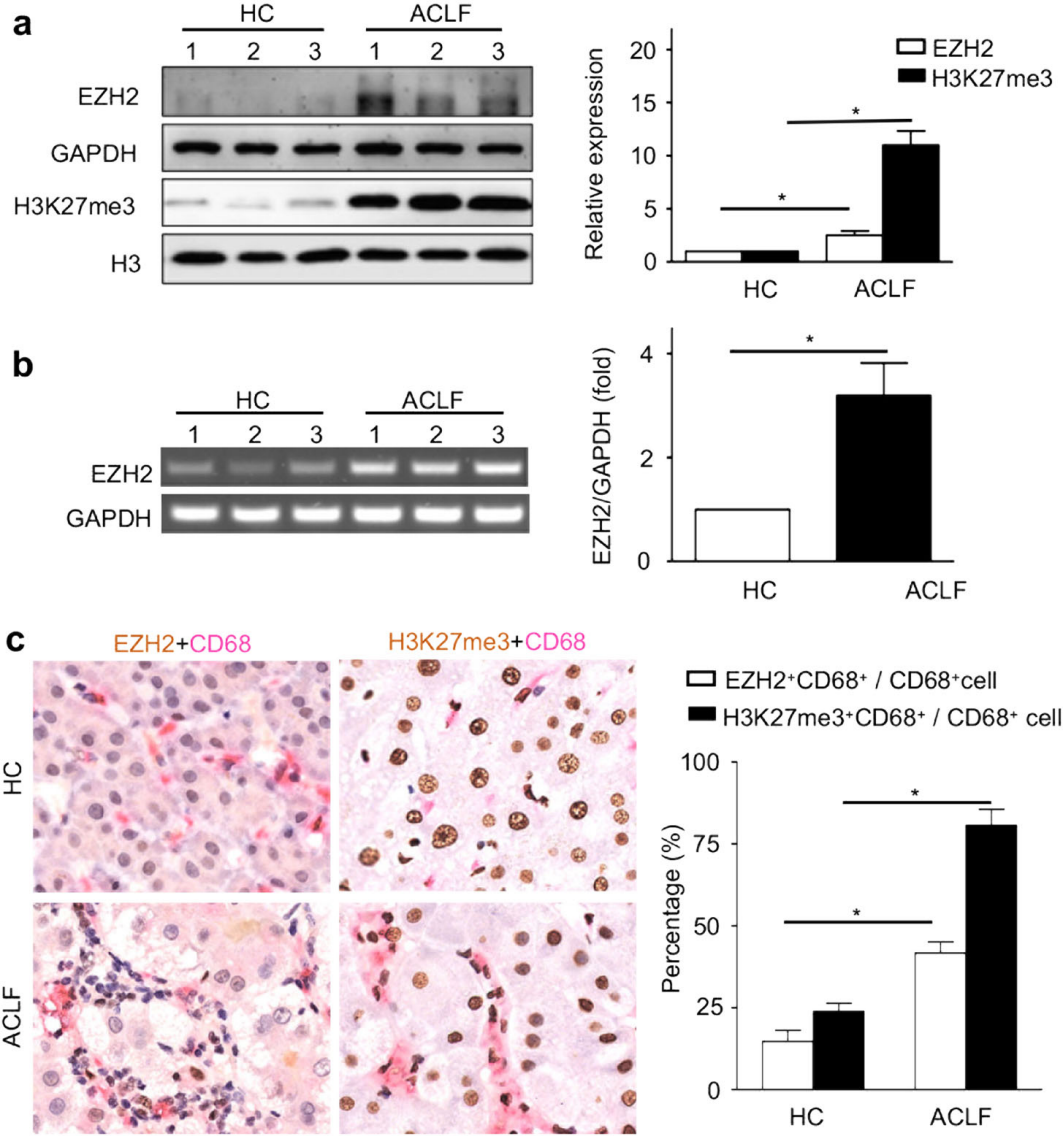
EZH2 and H3K27me3 production in ACLF patients. Comparison of EZH2 and H3K27me3 in PBMC between HC (*n* = 10) and ACLF (*n* = 10) were performed, using western blot (**a**) and RT-PCR (**b**). The bars represented bands density ratios of EZH2/GAPDH or H3K27me3/H3. *, *P* *<* 0.05. **c** It was illustrated that representative images of double staining immunohistochemistry [EZH2^43^ and CD68 (red) or H3K27me3^43^ and CD68 (red)] of liver from HC (*n* = 3) and ACLF (*n* = 3). Intrahepatic EZH2^+^CD68^+^ cells and H3K27me3^+^CD68^+^ cells were counted, and CD68^+^ cells were calculated using software ImagePro Plus 7.2. Percentage analysis of EZH2^+^CD68^+^ / CD68^+^ cells and H3K27me3^+^CD68^+^ / CD68^+^ cells was performed (*n* = 3). *, *P* *<* 0.05

ACLF is characterized by the presence of a precipitating event followed with chronic liver, leading to rapid progression of liver injury and ending in multi-organ dysfunction^16^. Although variation in definitions, the prevalence of ACLF is the highest among liver failure in hospitalized patients^17^. Recently numerous clinical studies demonstrate that immune response plays an important role in the occurrence and development of ACLF^2^. PBMC includes monocytes and lymphoblasts, and could reveal the immunity in many organs and various diseases^18^. To investigate the role of EZH2-mediated H3K27me3 in pathogenesis of liver failure, the levels of EZH2 and H3K27me3 in PBMC from ACLF patients and HC were determined using western blot. EZH2 or H3K27me3 in PBMC from ACLF patients was 2- or 10-fold higher than that from HC (*P* < 0.001) (Fig. 1a). The increased EZH2 in PBMC from ACLF patients was confirmed at mRNA level, using reverse transcription-PCR (Fig. 1b).

The hepatic EZH2 and H3K27me3 production during liver failure was determined in the liver sections from three ACLF patients, using immunohistochemistry. Intrahepatic EZH2^+^ cells were increased in the liver failure patients, some of them were closely related to CD68^+^ cells (Kupffer cells), accompanied with substantial increased infiltrating cells, compared to those from HC. Similarly, intrahepatic H3K27me3^+^ cells were observed in these liver failure patients (Fig. 1c). Interestingly, more than 2-fold Kupffer cells relating EZH2, as well as 3-fold relating H3K27me3, were detected in the liver failure patients, respectively (Fig. 1c).

### EZH2 and H3K27me3 are induced in TNF-mediated liver failure model

**Fig. 2:**
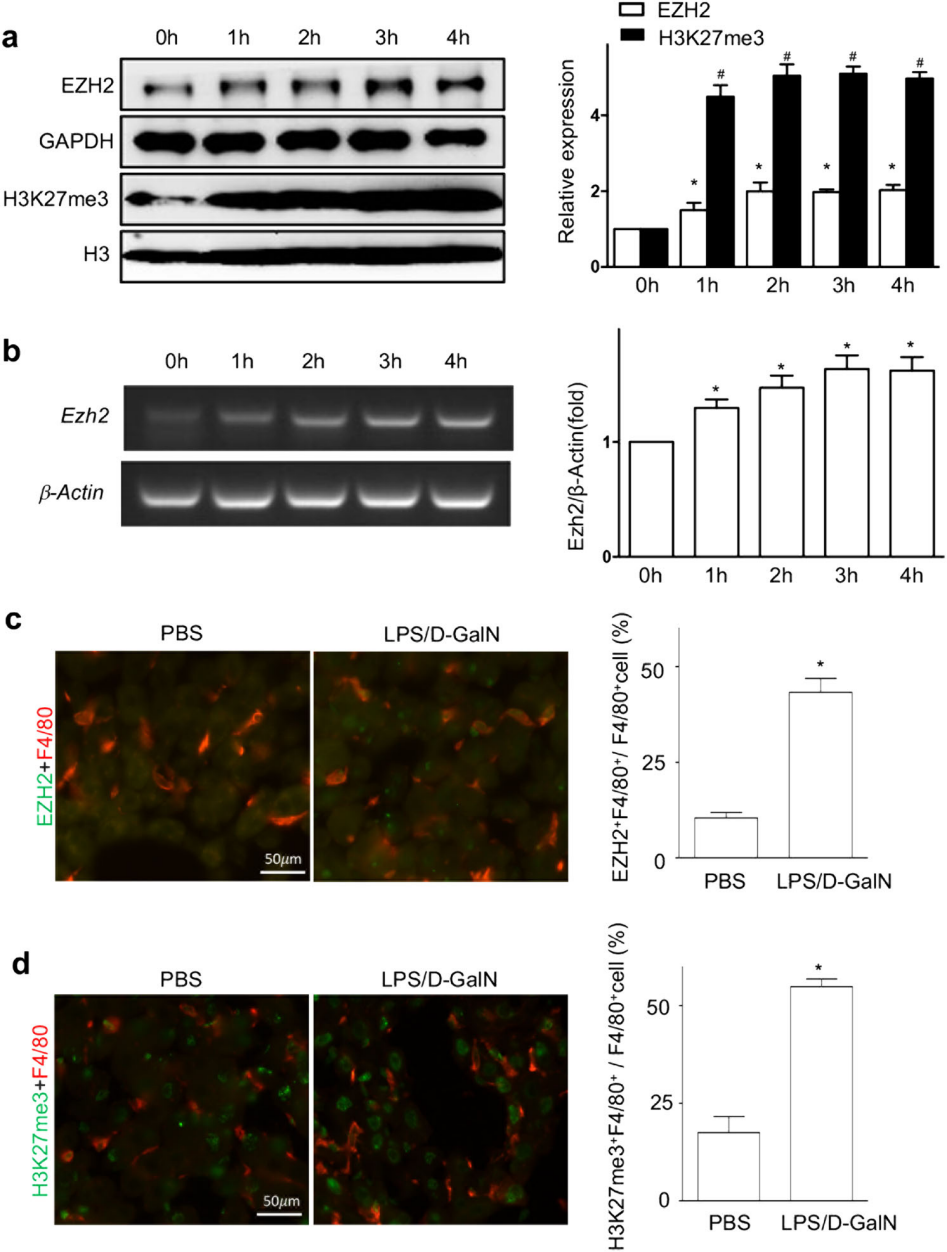
Hepatic EZH2 and H3K27me3 in liver failure mice. Production of EZH2 and H3K27me3 in Kupffer cells from LPS/D-GalN-induced liver failure mice was measured at the different time points, using western blot (**a**) and RT-PCR (**b**) (*n* = 5 per time point). The bars represent bands density ratios of EZH2/GAPDH or H3K27me3/H3. The data are shown as the mean ± SEM. *, *P* *<* 0.05 vs. EZH2 at 0 h; ^#^, *P* *<* 0.05 vs. H3K27me3 at 0 h. Two hours after intraperitoneal injection of PBS or LPS/D-GalN, mice in both groups were sacrificed and the liver tissues were collected. Representative images of immunofluorescence analysis showed co-staining of EZH2 (green) and F4/80 (red) (**c**), as well as H3K27me3 and F4/80 (**d**). Percentage analysis of EZH2^+^ F4/80^+^/F4/80^+^ cells and H3K27me3^+^ F4/80^+^/F4/80^+^ cells was performed. *, *P* *<* 0.05

ACLF is characterized as an acute deterioration based on chronic liver diseases, which is accompanied by excessive inflammatory response and frequently associated with endotoxemia^19^. To further determine the immunological role of EZH2 and H3K27me3 during the development of liver failure, the TNF-mediated liver injury models of lipopolysaccharide (LPS)/D-GalN-induced liver failure was established, which might be a reasonable tool to explore acute liver inflammation and mimic the acute deterioration period of ACLF. EZH2 and H3K27me3 in Kupffer cells from mice liver were increased significantly (*P* < 0.05) in liver failure mice following LPS/D-GalN injection (Fig. 2a). Consistently, EZH2 mRNA was also increased gradually with the induction (Fig. 2b).

There was a constitutive level of intrahepatic EZH2 and H3K27me3 in PBS-treated mice, with some F4/80^+^ Kupffer cells. After LPS/D-GalN injection, intrahepatic EZH2 was increased substantially, particularly in the highly inflamed region. The double immunofluorescence demonstrated that there were more EZH2^+^ Kupffer cells in high inflamed regions of LPS/D-GalN-injection mice than PBS-treated mice (*P* *<* 0.05) (Fig. 2c). Similar pattern of intrahepatic H3K27me3 and H3K27me^+^ Kupffer cells was observed (Fig. 2d). Significantly more Kupffer cells relating EZH2 and H3K27me3 were detected in the liver failure mice, respectively. Such data suggested that EZH2 and H3K27me3 are upregulated, some of Kupffer cells are able to produce EZH2 and H3K27me3 gradually during the progression of liver failure, which might be related to inflammation in liver tissues.

### Inhibition of H3K27me3 reduces mortality of liver failure and ameliorates liver injury

**Fig. 3:**
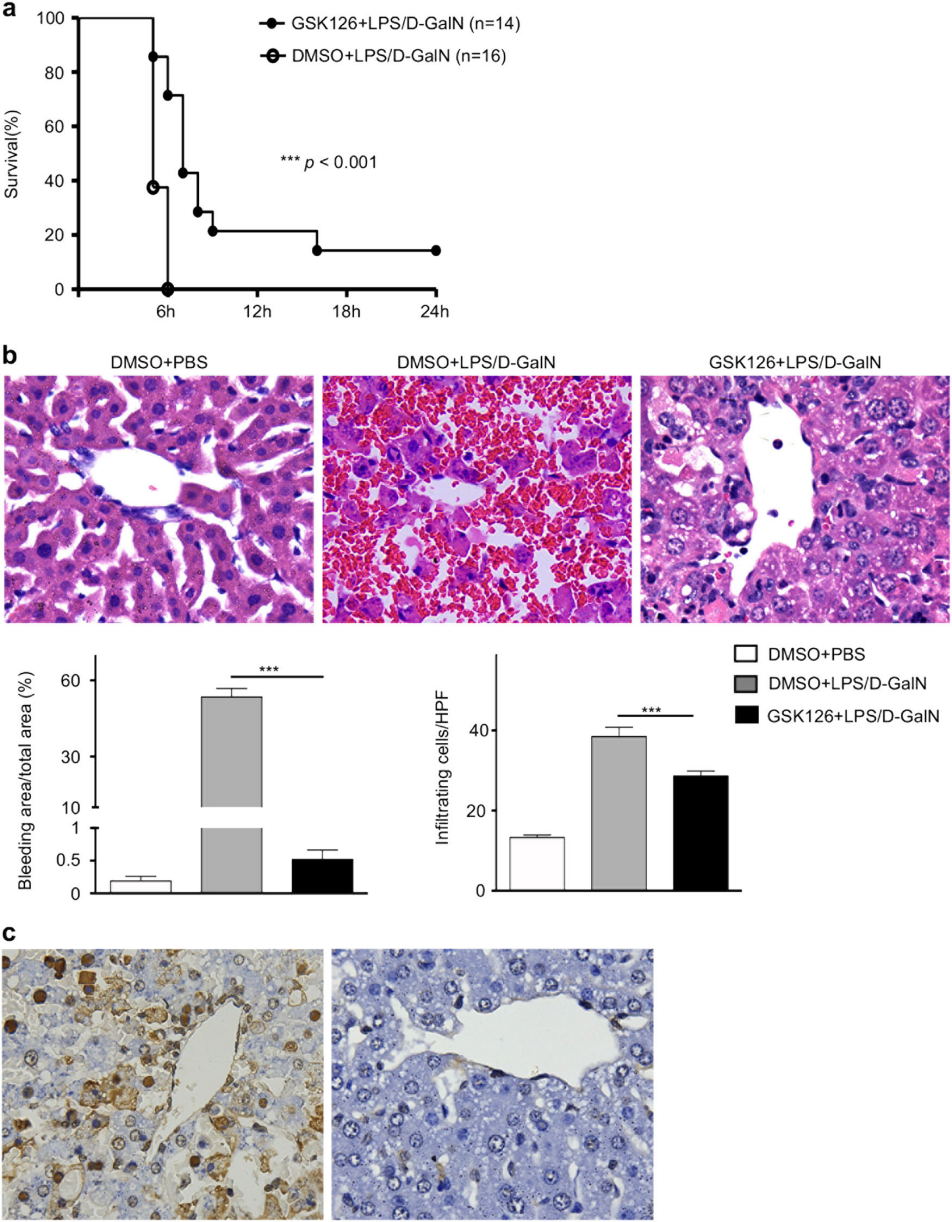
Mortality, liver histopathology in LPS/D-GalN-induced liver failure mice treated with/without GSK126. **a** It was presented that the mortality of LPS/D-GalN-induced liver failure mice treated with DMSO (*n* = 16) or GSK126 (*n* = 14). **b**, **c** In DMSO+PBS and GSK126+LPS/D-GalN groups, alive mice from were sacrificed at the observation end point of 24 h, and the liver were collected. In DMSO+LPS/D-GalN group, mice dead at 6 h with collection of the liver tissue. The livers from the three groups at the observation end point or dead point were subjected for HE staining, with bleeding and infiltration degree analysis. HPF high-power field; ***, *P* < 0.001 (**b**). The livers from the DMSO+LPS/D-GalN and GSK126+LPS/D-GalN groups were further subjected for TUNEL staining (**c**). Three independent experiments were performed with similar results

GSK126 is an inhibitor for EZH2 methyltransferase activity with high selectivity and efficacy^20^. Inhibition of EZH2-catalysed H3K27me3 in vivo was carried out using GSK126 to determine the function of EZH2 and H3K27me3 during liver failure in mice model. All of the vehicle (DMSO)-treated liver failure animals were died within 6 h post-LPS/D-GalN injection. In contrast, GSK126 treatment effectively prolonged survival time and improved survival rate (*P* < 0.001) (Fig. 3a). Accordingly, the histopathology showed that there was less intrahepatic bleeding and infiltrating cells in GSK126-treated group than that from the vehicle group at the end point of observation or dead point, respectively (*P* < 0.001) (Fig. 3b). Moreover, GSK126 also improved the hepatocyte necrosis/apoptosis in vivo (Fig. 3c). Such result was consistent with the survival rate during the development of liver failure.

### GSK126 suppresses proinflammatory cytokines in liver failure

Previous studies have reported that proinflammatory cytokines play critical roles during liver failure^4^. To explore the mechanism by which inhibiting methyltransferase activity of EZH2 protected mice from liver injury, proinflammatory cytokines during liver failure were examined.

**Fig. 4:**
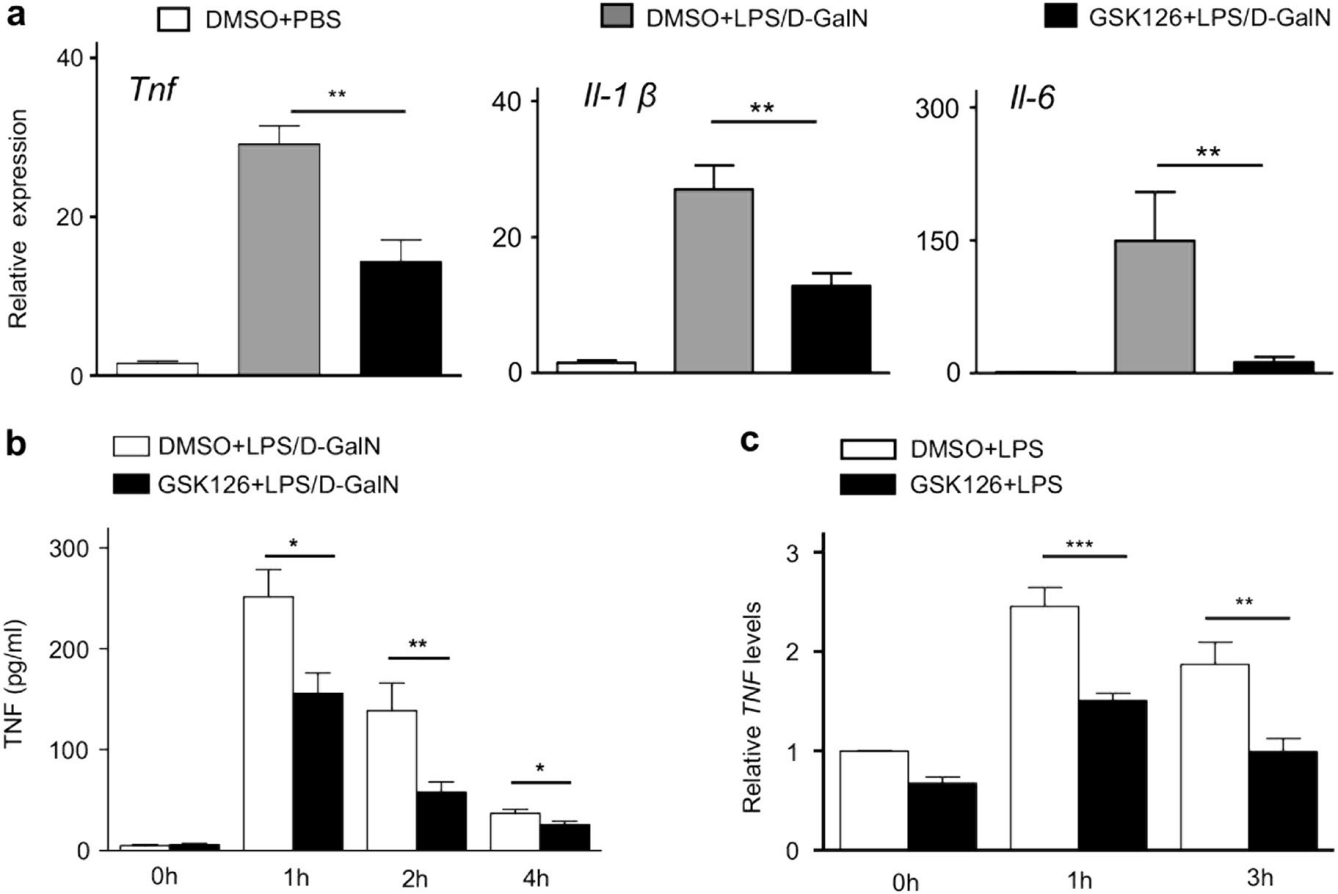
Proinflammatory cytokines production following addition of GSK126. **a** Hepatic *Tnf, Il-6* and *Il-1β* mRNA levels in the liver from the LPS/D-GalN-induced liver failure mice treated with or without GSK126 (*n* = 8 for each treatment). **b** Serum TNF production in LPS/D-GalN-induced liver failure mice treated with or without GSK126 (*n* = 8 for each treatment). **c** PBMC from human healthy volunteers were stimulated either GSK126 or vehicle (DMSO) 1 h prior to exposure of LPS (1 μg/ml) for the indicated times. *TNF* mRNA was measured using quantitative RT-PCR. *, *P* < 0.05; **, *P* < 0.01; ***, *P* < 0.001

Hepatic *Tnf, interleukin (Il)-1β*, and *Il-6* mRNA were determined from these induced liver failure mice with or without GSK126 pre-treatment. Hepatic *Tnf* mRNA was 15-fold increase in liver failure mice with the vehicle (DMSO) treatment, compared to that from non-liver failure animals (Fig. 4a). Increased *Tnf* mRNA was reduced by >40% in GSK126 pre-treated liver failure mice, compared to that from the vehicle-treated liver failure mice. Similar pattern was also detected in hepatic *Il-1β*, and *Il-6*, with the different treatments above (Fig. 4a). Serum TNF was increased substantially in liver failure mice with the vehicle (DMSO) treatment, peaked at 1 h, and gradually decreased till 4 h (Fig. 4b). However, GSK126 inhibited serum TNF substantially (*P* < 0.05), compared to that from the vehicle-treated liver failure mice.

To further investigate the effect of GSK126 on TNF in vitro, mRNA expression was determined in LPS-stimulated PBMC from HC treated with GSK126 or vehicle (DMSO). It was observed that TNF mRNA was increased 2.5- or 2-fold at 1 or 3 h, respectively, in the LPS-challenged PBMC with vehicle treatment (Fig. 4c). As expected, LPS-induced TNF expression in the PBMC was significantly inhibited 40% following GSK126 treatment. These data revealed that inhibiting EZH2-catalysed H3K27me3 by GSK126 reduced TNF and other important proinflammatory cytokines, both in vivo and in vitro.

### LPS/D-GalN causes reduction in H3K27me3 enrichment on *Tnf* promoter region

**Fig. 5:**
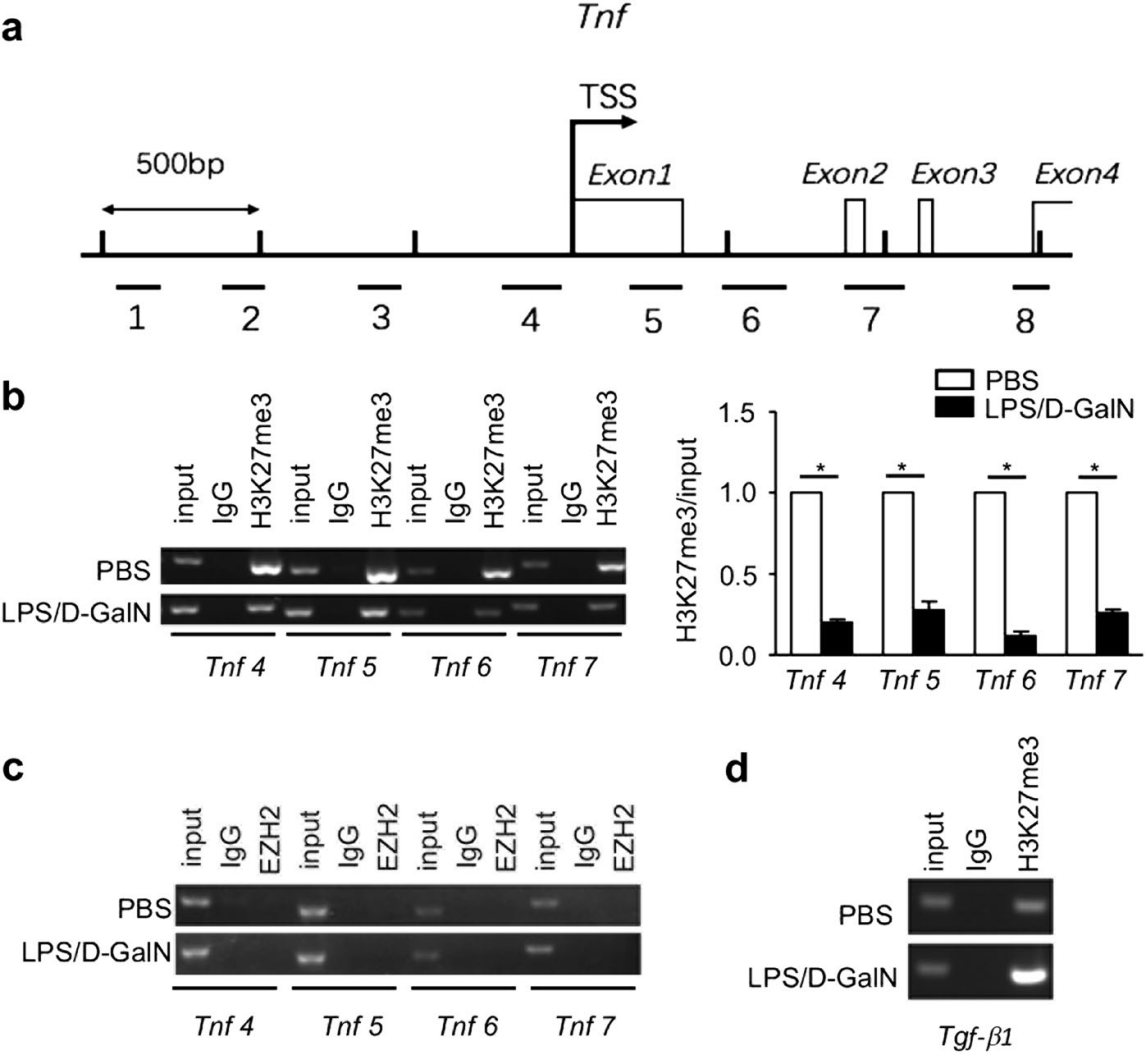
Changes of chromatin modifications on *Tnf* in Kupffer cells after liver failure induction. Mice were sacrificed in 1 h after injection of PBS or LPS/D-GalN, and the Kupffer cells from liver tissues were collected. **a** Genomic DNA fragments covering the −1.5 to +1.5 kb region for *Tnf* relative to the transcription start site for PCR analysis are indicated. ChIP assay was performed in the Kupffer cells to analyse H3K27me3 statuses (**b**) and EZH2 affinity (**c**) on the *Tnf* promoter region, and H3K27me3 statuses on *Tgf-β1* promoter region (**d**). The bars represented bands density ratios of H3K27me3/input enrichment. *, *P* < 0.05; **, *P* *<* 0.01; ***, *P* < 0.001

TNF is a major contributor to the inflammatory process in liver failure and secreted mainly by Kupffer cells during liver failure^21^. We further examined affinity of EZH2 and H3K27me3 on *Tnf* promoter in mice Kupffer cells. Eight pairs of primers were designed to amplify 100–150 bp around the regions indicated (Fig. 5a). Only four regions around the transcriptional start site of *Tnf* were detected with H3K27me3 enrichment (Fig. 5b), but not another four indicated regions (data not shown). Perhaps it was due to these four regions that were not responsible for the regulation of H3K27me3. Moreover, LPS/D-GalN injection in mice caused significant reduction in H3K27me3 enrichment on promoter region of *Tnf* in Kupffer cells (Fig. 5b), suggesting that the *Tnf* promoter was modified by the histone methylation during liver failure. Interestingly, no EZH2 recruitment was detected in these four particular regions, which match for the corresponding regions in H3K27me3 (Fig. 5c) from both control and liver failure mice. Furthermore, in the other four regions of the promoter region of *Tnf*, no EZH2 product was observed (data not shown). The data suggest that decreased H3K27me3 enrichment on *Tnf* promoter region contributes to *Tnf* transcription.

Various parenchymal cells are sensitive to TNF cytotoxicity, while transforming growth factor-β1 (TGF-β1) effectively attenuates the TNF-mediated necrosis/apoptosis^22,23^. Interestingly, H3K27me3 enrichment on promoter region of *Tgf-β1* was induced significantly upon liver failure (Fig. 5d).

### GSK126 attenuates nuclear factor kappa B (NF-κB) and protein kinase B (Akt) pathways

**Fig. 6:**
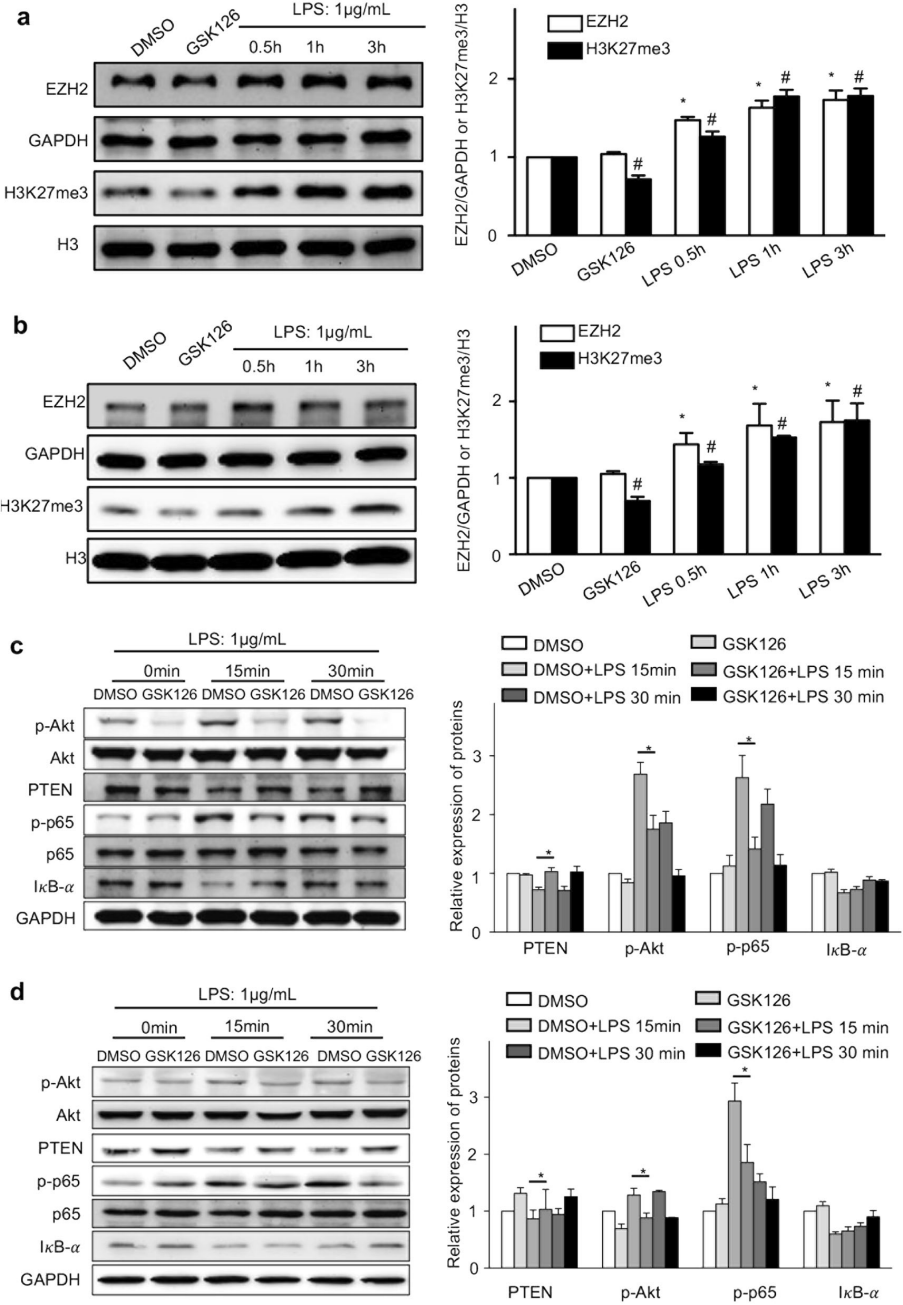
EZH2 and H3K27me3 facilitates TNF in immune cells via activation of NF-κB and PTEN-Akt pathways. Healthy mice splenic mononuclear cells (MNC) and human PBMC were obtained. MNC (**a**) and PBMC (**b**) were stimulated with either vehicle (DMSO) or GSK126 (10 nM/ml) for 1 h followed by addition of LPS (1 μg/ml) for the indicated times, respectively. The production of EZH2 or H3K27me3 was determined using western blot. The bars represented bands density ratios of EZH2/GAPDH or H3K27me3/H3. *, *P* < 0.05 vs. EZH2 of DMSO; ^#^, *P* < 0.05 vs. H3K27me3 of DMSO. MNC (**c**) and PBMC (**d**) were pre-treated with DMSO or GSK126 for 1 h, then stimulated with LPS (1 μg/ml) for the indicated times. Cell lysates were subjected for Western blot to detect the production of phosphorylated-Akt (p-Akt), IκB-α, PTEN, phosphorylated-p65 (p-p65), as well as total Akt and p65. The bars represented bands density ratios of p-Akt/Akt, PTEN/GAPDH, p-p65/p65 or IκB-α/GAPDH. *, *P* < 0.05

To further uncover the role of EZH2/H3K27me3 in regulating TNF in immune cells, EZH2 and H3K27me3 were measured in the healthy murine mononuclear cells and PBMC from HC following LPS stimulation (Fig. 6a, b). EZH2 or H3K27me3 were up-regulated 2- or 1.5-fold (*P* < 0.05) following LPS induction, respectively.

The efficacy of GSK126 in suppressing EZH2-catalysed H3K27me3 in the mononuclear cells were measured. EZH2 was not affected significantly, but H3K27me3 was decreased by >30% (Fig. 6a, b) following addition of GSK126. Such data suggest that GSK126 is efficient to interfere methylation of EZH2 without degradation of EZH2 per se.

Because LPS can activate NF-κB pathway in immune cells via TNF and other important cytokines, the related molecules indicating the activation of NF-κB pathway were further evaluated. Phosphorylation of p65 (p-p65) and degradation of NF-κB inhibitor-α (IκB-α) are markers for activation of the canonical NF-κB pathway^24^. As expected, p-p65 was significantly impaired by >40% in both murine splenic MNC and human PBMC with treatment of GSK126 upon LPS stimulation (Fig. 6c, d); whereas, degradation of IκB-α between DMSO and GSK126 treatment showed no significant differences. These data indicate that EHZ2 affects the immune cells activation by modulating the canonical NF-κB pathway via histone methylation.

It has been demonstrated that there is a direct involvement of Akt pathway in LPS-activated NF-κB pathways and other inflammatory molecules^8,25^. Phosphatase and tensin homolog deleted on chromosome ten (PTEN) is a critical upstream molecule for blockade of Akt pathway^26^. The indicators of Akt signalling pathways were observed in GSK126-treated murine MNC or PBMC from HC, compared to the vehicle-treated monocytes. As shown in Fig. 6c, d, phosphorylated-Akt was significantly suppressed, while PTEN was significantly induced following GSK126 treatment, suggesting that LPS-activated Akt pathways via reducing PTEN, which was inhibited by GSK126.

## Discussion

In the current study, there was a correlation between elevated EZH2/H3K27me3 production and upregulated proinflammatory cytokines during the development of liver failure. Furthermore, inhibitor GSK126 for EZH2-catalysed H3K27me3 ameliorated liver injury and improved survival in liver failure mice, via suppressing TNF and other indispensable proinflammatory cytokines. At the early phase of liver failure, EZH2/H3K27me3 promote the expression of proinflammatory cytokines, which was *via* the H3K27me3 enrichment, as well as NF-κB and Akt signalling pathways.

EZH2-mediated gene repression via H3K27me3 promotes immune response in hepatocellular carcinoma by regulation of CXC chemokine receptors^27^. Recently, EZH2/H3K27me3 has been shown to exert the essential role in maintained T cells differentiation and polarization for promoting immune response in alloimmunity^28^. This is in line with our data, showing that EZH2/H3K27me3 were upregulated with progression of liver injury and inflammation. It invites speculation that EZH2/H3K27me3 is involved in liver failure via regulating immune activation during liver failure.

As EZH2/H3K27me3 has been linked to many diseases^20,29^, modification of EZH2 becomes a hotspot in the development of pharmaceutical agents. 3-deazaneplanocin A (one of EZH2 inhibitors) reactivates the EZH2-silenced genes via degradation of the PRC2 complex, which causes some unexpected effects and is limited in clinical application^30^. Focusing on methyltransferase activity of EZH2 and the histone modification, GSK126 is selected by its highly selectivity in inhibiting EZH2 methyltransferase via competing with *S*-denosyl-methionine but without degradation of EZH2^20^. Such effect of GSK126 was also confirmed in human PBMC and murine splenic cells in the current study.

Epigenetic modification is crucial in the regulation of inflammatory cytokine production. Suberoylanilide hydroxamic acid, one of histone deacetylase inhibitors, shows satisfactory anti-inflammatory effects via reducing the production of proinflammatory cytokines in acute graft-versus-host disease^31^. These reports are consistent with our study, demonstrating that inhibition of EZH2-catalysed H3K27me3 using GSK126 significantly attenuated liver injury and impaired inflammatory cells infiltration, which might partially contribute to the reduced proinflammatory cytokines. Additionally, it was found that GSK126 reduces TNF expression and other proinflammatory cytokines directly in human PBMC in the current study. Collectively, our data suggest that EZH2 enhances some proinflammatory cytokines production upon stimulation during liver failure. Such data also provides compelling evidence to support potential clinical application of the targeting in EZH2 methyltransferase for liver failure.

Notably, using GSK126 inhibiting EZH2/H3K27me3 directly in vitro significantly reduced TNF in the current study, which raises an interesting question: how do EZH2 and H3K27me3 modulate the proinflammatory cytokines, especially TNF during liver failure. It was observed that H3K27me3 enrichment was reduced in *Tnf* promotor in Kupffer cells following liver failure, which is supported by others, showing *TNF* gene is regulated under H3K27me3 in breast cancer cells^32^. *Tnf* is regulated by coordinated actions of histone modifications^33^, our result confirmed that H3K27me3 is one of the predominate epigenetic components in *Tnf* at transcriptional level. On other hand, EZH2 acts as a gene activator in some cases via directly locating the promotor of target gene, in addition to the canonical role of EZH2 as a gene silencer^9^. Interestingly our data showed that no obvious affinity of EZH2 in *Tnf* promotor was observed in both normal and liver failure mice. Compared to directly binding on *Tnf* promotor as a transcription factor, EZH2 regulated TNF in liver failure might be largely due to its methylation activity and its downstream product (H3K27me3).

During liver failure, the global EZH2-catalysed H3K27me3 increased while its enrichment on *Tnf* promotor decreased. The explanation for such discrepancy might be that H3K27me3 enrichment increased on some other gene promotors to silencing the target genes. It was further investigated H3K27me3 enrichment on promotor of other genes, especially *Tgf-β1* which protects various parenchymal cells from TNF cytotoxicity^22,23^. It is consistent with Mondal et al., indicating EZH2/H3K27me3 regulated TGF-β1 and the related genes^34^. We speculated that *Tgf-β1* represented one of many genes which were silenced via the H3K27me3 enrichment on their promotors during liver failure. It might be an epigenetic mechanism in which EZH2/H3K27me3 promoted TNF-mediated liver failure and GSK126 could improve the liver inflammation.

Finally, the linkage between EZH2/H3K27me3 and other possible signalling pathway(s) in immunity was also explored. EZH2-mediated gene silence promotes Toll-like receptors (TLRs) *via* suppressing LRRC33 in LPS-stimulated macrophages in vivo and in vitro^35^. TLR pathways may affect the induction of many immediate early genes involved in the proinflammatory cytokines during the liver failure^20,32^. Furthermore, NF-κB pathway, an important part of TLRs pathways, plays pivotal roles in TNF^36^. In addition, Akt signalling pathway is closely related to immune regulation following activation of TLRs pathway^37^. This is further supporting our current study, showing GSK126 suppresses LPS-activated signalling pathways and subsequently promoting inflammatory cytokines.

Overall, our data suggested that EZH2/H3K27me3 promoted the development of liver failure, which was ameliorated using GSK126 via modulation of TNF and overall inflammation. Moreover, TNF was manipulated with the histone modification of H3K27me3, as well as the downstream signalling pathways of NF-κB and Akt. Thus, our data provided compelling evidence to support potential clinical application of pharmacological targeting of epigenetic modifications, specifically for treatment of ACLF.

## Materials and methods

### Human subjects

A total of 20 subjects were enrolled between June 2016 to May 2017 in Department of Infectious Diseases, Ruijin Hospital, Shanghai, China, including hepatitis B virus-related acute-on-chronic liver failure patients^16^ (*n* = 10) and healthy controls (HC, *n* = 10). Characterization was based on *Asian Pacific Association for the Study* of the Liver criteria^16^. The study was approved from *the Human Ethics Committee, Ruijin Hospital, Shanghai Jiao Tong University School of Medicine*. The written consent form was obtained from each subject.

### Induction of liver failure in mice

C57BL/6 female mice, 10-week old with body weight of 18–20 g, were injected intraperitoneally with 10 μg/kg of LPS (Sigma-Aldrich, St. Louis, MO, USA) and 700 mg/kg D-galactosamine (D-GalN) (Sigma-Aldrich, St. Louis, MO, USA) dissolved in 200 μl PBS for induction of liver failure. For the indicated experiments, these mice were pre-treated with intraperitoneal injection of vehicle (5% DMSO) or 10 mg/kg GSK126 (Selleckchem, Houston, TX, USA) dissolved in 200 μl 5% DMSO for 2 h, and then subjected for liver failure induction. The harvested serum and liver tissues were analysed at the indicated time points. Kupffer cells isolation were also perform as described in supplementary materials. All animal studies were approved by *the Institutional Animal Care and Use Committees of Ruijin Hospital*.

### Western blot, reverse transcription-PCR and cytokine analysis

The liver tissue and cell lysis were subjected for Western blot to detect protein products^38^, and exacted for total RNA and then subjected for reverse transcription-PCR^39^. Murine serum TNF was also analysed using ELISA (supplementary materials). More details were described in the supplementary materials.

### Immunostaining

Double staining of EZH2 and CD68 was performed as described^40^. Briefly, sections (4 μm) were heat-induced epitope retrieval, using sodium citrate buffer (pH 6) for 5 min. The sections were incubated with primary antibodies mixture of rabbit anti-EZH2 antibody (1:200; Cell Signalling Technology) and mouse anti-CD68 antibody (1:600; Abcam) for 8 h incubation at 4 °C. Subsequently, antibodies mixture of peroxidase AffiniPure goat anti-rabbit IgG^41^ and alkaline phosphatase AffiniPure goat anti-mouse IgG^41^ were followed for 30 min at room temperature. Finally, EZH2 or CD68 was detected, using 3,3′-diaminobenzidine (DAB, Sigma-Aldrich) or alkaline phosphatase-red (Shanghai ZuochengBio). Similar double staining method was applied for H3K27me3 (rabbit anti-H3K27me3 antibody, 1:200; Cell Signalling Technology) and CD68 staining (as described above).

Frozen sections (8 μm) were applied in the murine tissues for co-localization of F4/80 with EZH2 or H3K27me3 using double immunofluorescence. Briefly, incubation with blocking solution (containing goat serum) at room temperature for 1 h was performed, followed by an overnight incubation at 4 °C with the mixture of anti-EZH2 antibody (1:200; Cell Signalling Technology) and anti-F4/80 antibody (1:100; Bio-Rad), or anti-H3K27me3 antibody (1:600; Cell Signalling Technology) and anti-F4/80 antibody (1:100; Bio-Rad), respectively. Incubation with mixtures of Alexa Fluor 488-conjugated secondary antibody (1:200; Invitrogen) and Alexa Fluor 555-conjugated secondary antibody (1:200; Invitrogen) for 1 h at room temperature in the dark was performed, followed by a 5 min incubation of DAPI at room temperature in the dark.

### Histological analysis

Liver tissues were collected and then fixed in 10% formalin and embedded in paraffin were subjected for hematoxylin–eosin staining as previously described^38^.

Using image analysis software ImagePro Plus 7.2 (Diagnostic Instruments), the intrahepatic bleeding was determined, using necrosis area and bleeding area versus total hepatic are in the field, respectively. The infiltrating cells were counted, as described^42^.

The sections were subjected for terminal deoxynucleotidyl transferase dUTP nick end labeling (TUNEL) assays using colorimetric TUNEL apoptosis assay kit (Beyotime) according to the instruction from the manufacturer. It was detected using DAB for visualization.

### Chromatin immunoprecipitation (ChIP) assays

ChIP analysis was applied for these isolated Kupffer cells using EZ-Magna ChIP^TM^ A kit (Millipore), following the instructions from the manufacturer. The antibodies of EZH2 and H3K27me3 used in the ChIP assay were both purchased from Cell Signalling Technology. The purified DNA fragments were amplified for PCR. The used primers were listed in the supplementary materials.

### Cell preparation and stimulation

Peripheral blood mononuclear cells (PBMC) from HC human subjects and splenic mononuclear cells (MNC) from normal mice were obtained using Ficoll density gradient separation, and maintained in complete medium which contained 10% foetal bovine serum and RPMI1640 medium (Gibco). Then these cells were plated in 12-well plates as a density of 1 × 10^6^ cells/ml with LPS (1 μg/ml). For the indicated experiments, these cells were pre-treated with 2 μl/ml DMSO or 10 nM/ml GSK126 (dissolved in DMSO) 1 h prior to LPS stimulation.

### Statistical analysis

All results are described as means ± SEM, which were further calculated by two-tailed, unpaired or paired Student’s *t* tests for significant differences. In addition, log-rank tests were performed for survival analysis. All experiments had been repeated for at least three times. *P* < 0.05 was set as statistical significance. *, *P* < 0.05; **, *P* < 0.01; ***, *P* < 0.001.

## Electronic supplementary material

supplementary figure legends
