# Supplementary material for: Enhancer of zeste homolog 2-catalysed H3K27 trimethylation plays a key role in acute-on-chronic liver failure via TNF-mediated pathway
Source: Cell Death Dis. 2018 May 22;9(6):590. doi: 10.1038/s41419-018-0670-2 (PMC5964223; doi:10.1038/s41419-018-0670-2)
Supplement: Supplementary file 1 — supplementary figure legends [file 41419_2018_670_MOESM1_ESM.docx]

**Supplementary methods**

**Enhancer of zeste homolog 2-catalysed H3K27 trimethylation plays a key role in liver failure via TNF-mediated pathway**

Tianhui Zhou^1,#^; Ye Sun^1,#^; Yongsen Ding^1^; Rongkun Yin^1^; Ziqiang Li^1^; Qing Xie^1^; Shisan Bao ^2, *^ and Wei Cai^1, *^

**Kupffer Cells Isolation**

Liver failure or the control mice were sacrificed at the indicated time points. Livers were perfused with EGTA buffer and Gey’s balanced salt solution (MACGENE) containing collagenase (Roche) *via* portal vein. Kupffer cells were isolated by gradient centrifugation using 30%/70% Percoll (GE Healthcare Life Science) solution, and further purified, using CD11b microbeads (Miltenyi Biotec, Auburn, CA, USA) according to the instruction. These sorted Kupffer cells were confirmed using flow cytometry with purity more than 98%.

**Western Blot**

Western blotting was performed as previously described (1). In brief, cell lysis was exacted by RIPA (Pierce) containing protease and phosphatase inhibitors (Roche Diagnostics, Mannheim, Germany), and separated by 10% SDS-PAGE after denaturation. The primary antibodies involved were as follows: EZH2 (1:2000, Cell Signalling Technology), H3K27me3 (1:2000, Cell Signalling Technology), H3 (1:3000, Cell Signalling Technology), protein kinase B (Akt) (1:2000, Cell Signalling Technology), phosphorylation of Akt (1:2000, Cell Signalling Technology), phosphatase and tensin homolog deleted on chromosome ten (PTEN) (1:1000, Cell Signalling Technology), nuclear factor kappa B (NF-κB) inhibitor α (IκB-α) (1:1000, Cell Signalling Technology), phosphorylation of p65 (p-p65) (1:1000, Cell Signalling Technology), p65 (1:1000, Cell Signalling Technology), GAPDH (1:5000, Santa Cruz).

**Reverse Transcription-PCR**

RT-PCR were performed, as previously described (2). In brief, the tissue and cell lysis was exacted for total RNA using TRIzol (Invitrogen), and RNA was reverse transcribed for cDNA using PrimeScript RT Master Mix. The used primer pairs used were listed as follows (5’-3’):

h*GAPDH* forword: GGATTTGGTCGTATTGGG, reverse: GGAAGATGGTGATGGGATT;

h*EZH2* forword: GAGTTGGTGAATGCCCTTGG, reverse: TGCTGTGCCCTTATCTGGAA;

m*β*-actin forword: TTCCAGCCTTCCTTCTTGG, reverse: TGTTGGCATAGAGGTCTTTACGG;

m*Ezh2* forword: CGAATAACAGTAGCAGACCCAG,

reverse: TGTTTGACACCGAGAATTTGCTT.

**Cytokine Analysis**

Murine serum TNF was assessed using ELISA kits (R&D Systems, Minneapolis, MN, USA), according to its manufacturer instructions.

Murine hepatic *Tnf, interleukin (Il)-1β, Il-6* and human *TNF* in PBMC were assessed using quantitative RT-PCR, (TAKARA BIO INC, Otsu, Shiga, Japan). The used primer pairs were listed as follows (5’-3’):

h*GAPDH* forword: GGATTTGGTCGTATTGGG, reverse: GGAAGATGGTGATGGGATT;

h*TNF* forword: ACACCATGAGCACTGAAAGC, reverse: CGATCAGGAAGGAGAAGAGG;

m*β-actin* forword: TTCCAGCCTTCCTTCTTGG, reverse: TGTTGGCATAGAGGTCTTTACGG;

m*Tnf* forword: GGTCTGGGCCATAGAACTGA, reverse: CAGCCTCTTCTCATTCCTGC;

m*Il-1β* forword: CTGGTACATCAGCACCTCAC, reverse: AGAAACAGTCCAGCCCATAC;

m*Il-6* forword: TGTATGAACAACGATGATGCACTT,

reverse: ACTCTGGCTTTGTCTTTCTTGTTATCT.

**Chromatin Immunoprecipitation (ChIP) Assays**

ChIP analysis was applied for these isolated Kupffer cells using EZ-Magna ChIP^TM^ A kit (Millipore), following the instructions from the manufacturer. The antibodies of EZH2 and H3K27me3 used in the ChIP assay were both purchased from Cell Signalling Technology. The purified DNA fragments were amplified for PCR. The used primers were listed as follows (5’-3’):

*Tnf 1* forward: GGAGAAGGCTTGTGAGGTCC, reverse: AAGGCAGAGCAGCTTGAGAG;

*Tnf 2* forward: TTAAGGAGTGGAGCAGGGGA, reverse: GGTCTCCTCCGGCAGTTAAG;

*Tnf 3* forward: GGGAACAGGGGAAGGTTGAC, reverse: ATGGGTCCTGTCTGAGGTGA;

*Tnf 4* forward: ATGCCTGGGTGTCCCCAACTT, reverse: GTGCAGACGGCCGCCTTTATA;

*Tnf 5* forward: ACAGAAAGCATGATCCGCGACG, reverse: GGGGACCGATCACCCCGAAGT;

*Tnf 6* forward: GAAGATGAAGGGGAGATAACG, reverse: GGAGAAGCCTCCCGGCTGATT;

*Tnf 7* forward: TCTTAACTAACCTCCTTTTCCTAC, reverse: CCCCTTTCCTCCCAAACCAAA;

*Tnf 8* forward: GGGTGACACTGACTCAATCC, reverse: GTCTTTGAGATCCATGCCGTTG;

*Tgf-β1* forward: CGCCTAGGTCCCCACTTCTA, reverse: GGAGCACTACTAAAGCCGGT.

**REFERENCE**

1. Zhou T, Jin M, Ding Y, et al: Hepatitis B virus dampens autophagy maturation via negative regulation of Rab7 expression. *Biosci Trends* 2016; 10:244-250.

2. Cai W, Du A, Feng K, et al: Adenylyl cyclase 6 activation negatively regulates TLR4 signaling through lipid raft-mediated endocytosis. *J Immunol* 2013; 191:6093-6100.
